# Bittersweet: relevant amounts of the common sweet food additive, glycerol, accelerate the growth of PC3 human prostate cancer xenografts

**DOI:** 10.1186/s13104-022-05990-9

**Published:** 2022-03-10

**Authors:** Ariel DeGuzman, Mary Y. Lorenson, Ameae M. Walker

**Affiliations:** Division of Biomedical Sciences, School of Medicine, University of California, Riverside, CA 92521 USA

**Keywords:** Tumor promoter, Inadvertent consumption, Diet, e-cigarettes

## Abstract

**Objective:**

In a study of potential prostate cancer therapeutics, glycerol was used to increase the density of one solution. Glycerol alone was therefore one of the controls. Tumors of human PC3 castrate-resistant prostate cancer cells were initiated in male nude mice and grown for 12 days. Mice were then sorted such that mean tumor weights were the same in each group, and osmotic minipumps delivering 0.25 µL/h of either saline or glycerol were then implanted subcutaneously.

**Results:**

Contrary to our initial assumption that glycerol would be without effect, tumors grew more rapidly in the glycerol group such that tumors were twice the size of those in the saline group after 4 weeks. Given the dose delivered, analysis of the literature suggests this effect was not via the conversion of glycerol to glucose but possibly via a reduction in oxidative damage in the growing tumor. Our data demonstrate that amounts of glycerol that could reasonably be derived from the diet promote the growth of these tumors. Given the increasing use of glycerol in foods and beverages, we present these data to stimulate interest in an epidemiological study in the human population examining glycerol consumption and the aggressiveness of prostate cancer.

## Introduction

Grocery stores today offer a variety of prepared foods, beverages and mixes, the plusses of which include longer shelf life and ease of meal preparation. One additive that increases shelf life of a wide variety of products is glycerol, also known as glycerin or glycerine. Glycerol is an additive in processed meats, cheeses, dairy drinks, sweet beverages, beer, white wine, cakes, confectionaries, dietetic foods and dried fruits and nuts. It is sweet and viscous, providing both taste and smooth texture in one additive. It is also a humectant, thereby reducing product dehydration upon storage. In addition, it stabilizes emulsions, increasing the shelf-life of many items [1]. In some brands of e-cigarettes, glycerol is a major component of the flavored liquid used to create the vapor that acts as a vehicle for nicotine inhalation [2].

Glycerol is a natural compound and, as an additive, is considered safe and non-toxic by the United States Food and Drug Administration [3]. Food additives are also evaluated regularly for safety by a Joint Food and Agriculture Organization/World Health Organization Expert Committee [4]. Glycerol in the human and mouse body is derived from the hydrolysis of triglycerides in the digestive system [5] or from hydrolysis of triglycerides in adipose tissue during fasting [6]. It is distributed in extracellular fluids, including plasma, and above a certain threshold is excreted by the kidneys. It is phosphorylated to glycerol-3-phosphate (G3P) by glycerol kinase in the liver (80–90%) and kidneys (10–20%) and can be metabolized to glucose [7]. Normal serum levels of glycerol in adult humans range from 0.05 to 0.1 mmol/L, although values of 0.07 mmol/L and above correlate with obesity and insulin resistance [6].

Prostate cancer is the second leading cause of cancer deaths in men in the United States and 1 in 8 men will develop prostate cancer at some point in their life [8]. While a majority of prostate cancers initially respond to androgen deprivation therapy, most will eventually progress to a castrate-resistant state. PC3 cells are a human prostate cancer cell line, derived from a bone metastasis of stage IV castrate-resistant adenocarcinoma, frequently used as a xenograft model in immunocompromised mice to test new therapeutics aimed at late-stage disease. This brief report documents a serendipitous finding resulting from the use of glycerol to increase viscosity of a test therapeutic. Comparison of the two controls used in the study demonstrated that small quantities of glycerol contributed to the growth of PC3 prostate cancer cell xenografts, causing a remarkable doubling of tumor size *versus* saline over a 4-week period.

## Main text

### Materials and methods

PC3 human prostate cancer cells were freshly obtained from American Type Culture Collection (ATCC CRL-1435, Manassas, VA) to ensure their authenticity. The stock was amplified by culture in Modified Eagle’s medium supplemented with 10% fetal bovine serum. When ready to seed the tumors, trypsinized PC3 cells were washed in Dulbecco’s phosphate buffered saline (DPBS) and then suspended in 50% Matrigel (Becton, Dickinson and Company, Franklin Lakes, NJ) in DPBS. Six-week-old male Foxn1 (Nu/Nu) Nude (Crl:NU-Foxn1nu, Charles River) mice were allowed to acclimate to their surroundings in a barrier facility for 2 weeks before experimentation. The mice were housed, 5 to a microisolator cage, with sterile paper chip bedding, and 12 h light–dark cycles, and were provided with sterile chow and water ad libitum. The cages were enriched with sterile nestlet domes. At eight weeks of age, mice were inoculated with 5 × 10^6^ PC3 cells, subcutaneously in the left flank (day 0), alternating between groups. On day 12, mice were assigned to each treatment group such that mean tumor sizes were the same and variance was <  ± 10%. On this day (between 8 and 10 am), Alzet osmotic minipumps (#2004, Alza, Palo Alto, CA), delivering 0.25 µL/h of either saline or glycerol (> 99.5%, Sigma-Aldrich, St Louis, MO; 1.26 g/mL), were implanted subcutaneously in the interscapular area, again alternating groups. Because implantation takes only a few minutes, isoflurane anesthesia using a precision vaporizer was used such that post-surgical recovery was rapid. Implantation was performed in a laminar flow hood under aseptic conditions in a vivarium procedure room. Bupivacaine affords up to 8 h of post-surgical analgesia (7 mg/kg subcutaneously) and therefore was the analgesic of choice. Neosporin antibiotic ointment was applied externally to the wound-clipped incision. The health of the mice was subsequently checked daily, and wound clips were removed at days 17–19, according to incision healing. Animal weights and tumor sizes were assessed twice weekly. Tumor size was assessed by fine caliper measurements by technicians blind to the treatment groups. All mice were euthanized at day 44 by decapitation (to recover trunk blood) under low stress conditions (gentle handling, no sight or smell of procedure applied to previous animal) when the mean tumor weight in one group reached ~ 1500 mg. All procedures involving animals were approved by the University of California, Riverside Institutional Animal Care and Use Committee (Protocol # A20190048E for AMW). Calculated tumor weight in these loose-skinned mice was based on the formula 1/2 (length × width × height) and an assumption that the tumor has the density of water (1 g/cm^3^). Because this is a calculated value, the ordinate on the tumor growth graph is labeled as relative tumor size (mg) to indicate that the mg measurement is not an actual weighed value. Data are presented as the mean ± SEM of 5 animals per group. This number was based on the expectation that there would be no effect of glycerol and hence that both control groups in the larger study could likely be combined. Power analysis for the larger study, which study had to be discarded because of the glycerol effect, recommended a group size of 10 because of the therapeutic combination and dose–response aspects being investigated. Given normal variation and the simple saline *versus* glycerol, a group size of 5 would have been sufficient. Tumor growth rates in each group were compared by simple linear regression with 95% confidence intervals. Graphpad software (San Diego, CA) was used for all statistical analyses and production of the graphs.

### Results

**Fig. 1:**
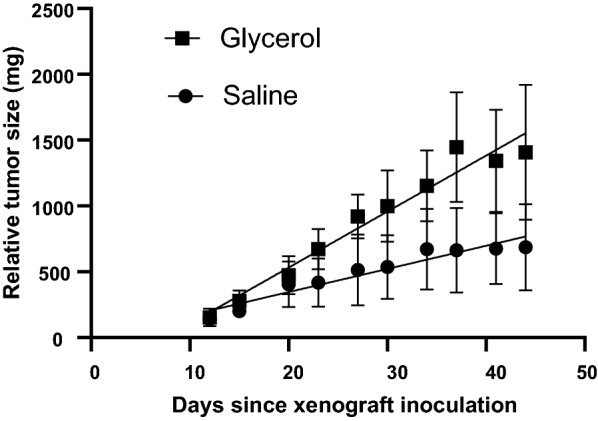
Tumor growth in male mice receiving either glycerol or saline. Test substances (glycerol at 0.315 mg/h or saline) were administered via osmotic minipump beginning on day 12 after xenograft inoculation. Points are mean ± SEM. *n* = 5 mice per group

**Fig. 2:**
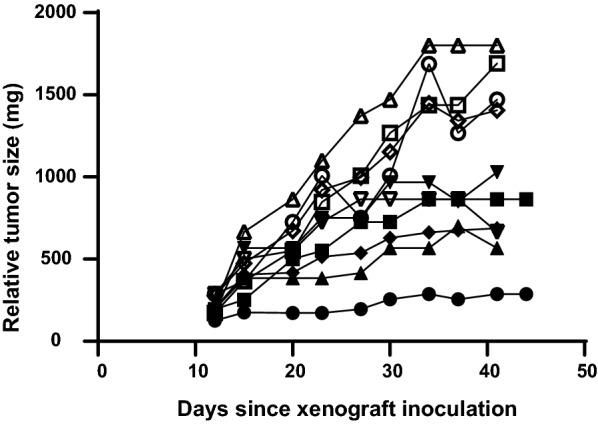
Tumor growth in individual mice. This figure uses the same mice as in Fig. 1. Open symbols are animals given glycerol and closed symbols are the saline controls

Mice receiving glycerol at a dose of 0.315 mg/h (0.25µL × 1.26 g/mL) showed a steady, higher rate of PC3 xenograft growth *versus* those receiving saline (Fig. 1, *p* < 0.0001) such that the mean calculated weight in the glycerol group was twice that in the saline group at day 44 (1406 v 687 mg), at which time the study was terminated due to tumor burdens. Day 44 was 32 days after pump implantation. Tumor growth rates in individual animals are presented as Fig. 2.

**Fig. 3:**
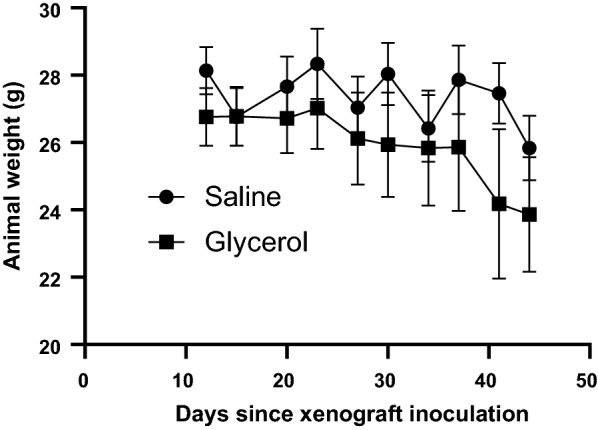
Body weights of mice during xenograft growth. Test substances (glycerol at 0.315 mg/h or saline) were administered via osmotic minipump beginning on day 12 after xenograft inoculation. Points are mean ± SEM. *n* = 5 mice per group

Mice in the saline group maintained their body weight (bw) throughout most of the trial, whereas those in the glycerol group lost weight over time (Fig. 3, *p* = 0.0008), most likely as a result of increased tumor burden and resultant cachexia despite additional calories from the glycerol.

### Discussion

These results were a serendipitous finding from controls in a study where glycerol was being used to increase the viscosity of a solution delivered by Alzet minipump. The original assumption was that as a natural compound being delivered at a low dose, it would be a component of no consequence. However, delivery of glycerol caused the tumors to grow much more rapidly.

Our first consideration as to mechanism was the conversion of glycerol to glucose and therefore the provision of extra glucose to the growing tumor. However, this explanation does not seem likely because the dose of glycerol was far below that necessary to result in an elevation of blood glucose after glycerol metabolism in the liver [9]. For example, administration of glycerol to normal mice at 2 g/kg (50 mg/25 g mouse) intraperitoneally results in a peak glucose level of 60 mg/dL at 45 min [9]. This dose is ~ 150 fold the dose provided per hour in our study and the resultant glucose level was still only 60 mg/dL. Even with continuous infusion rather than a single injection, it therefore seems unlikely that the concentration of glycerol in our study would have significantly increased circulating glucose. An alternative mechanism may be suggested by a recent publication showing that aggressive human prostate cancers have increased expression of G3P phosphatase (G3PP) [10]. This G3PP converts G3P to free glycerol (akin to our administered glycerol), which in turn reduces toxic oxidative stress within the tumor, thereby allowing greater tumor growth.

Probably the best illustration of how the dose in our study compares to one potential dietary source is the documentation that Duvel beer contains 227 mg/dL glycerol (white wine can contain 469 mg/dL) [11]. Assuming consumption of 500 mL of beer during a 1-h period, this would amount to a dose of 15.1 mg/kg bw/h in a 75 kg male. Rounding to a 25 g mouse, the dose used in our study was 16.4 mg/kg bw/h. These calculations suggest that the concentration of glycerol in foods and beverages could contribute to more rapid progression of prostate cancer. Exposure would be increased by the consumption of some of the many other glycerol-containing foods and might also be increased with absorption of glycerol from e-cigarette vapor [2] and the multitude of glycerol-containing self-care/cosmetic products [12].

Given the incidence of prostate cancer, any aspect of life that potentially increases the risk of dying from this disease is of concern, especially when the forecast is for the use of glycerol to markedly increase [13]. In addition, given the elevation in circulating glycerol in obesity and the quantities of glycerol in some alcoholic beverages, glycerol may be an important link between obesity, alcohol and aggressive prostate cancer [14].

While glycerol is not a carcinogen, our serendipitous finding together with the recent work of Lounis et al. showing increased expression of G3PP in aggressive prostate cancer [10] suggests glycerol should be evaluated as a potential tumor promoter, at least in prostate cancer.

## Limitations

Since our major research focus is the development of new therapeutics for castrate-resistant prostate cancer, initial in vivo testing is usually limited to tumors derived from one appropriate cell line. Unknown therefore is whether this response to glycerol is peculiar to PC3 cells or representative of castrate-resistant lines and patient-derived xenografts in general. Also unknown is whether glycerol may enhance the growth of other types of cancer.

## Data Availability

All raw data are available to other qualified investigators upon request to corresponding author.

## Abbreviations

bw: Body weight
DPBS: Dulbecco’s phosphate buffered saline
G3P: Glycerol-3-phosphate
G3PP: Glycerol-3-phosphate phosphatase
